# Enhanced efficiency of melatonin by stepwise-targeting strategy for acute lung injury

**DOI:** 10.3389/fbioe.2022.970743

**Published:** 2022-09-07

**Authors:** Hongbo Wang, Jing Li, Jianbo Jin, Jingbo Hu, Chunlin Yang

**Affiliations:** ^1^ Department of Pharmacy, Ningbo University Affiliated Yangming Hospital, Yuyao, China; ^2^ Faculty of Materials Science and Chemical Engineering, Ningbo University, Ningbo, China

**Keywords:** acute lung injury, vascular endothelium, oxidative stress, mitochondria, lung targeting, melatonin

## Abstract

Oxidative stress plays a key role in the progress of acute lung injury (ALI), which is an acute, progressive respiratory failure characterized by alveolar capillary injury caused by various external and internal factors other than cardiogenic factors. Pulmonary vascular endothelial cells are the main target cells during ALI, and therefore the mitochondrial targeting antioxidant derivative triphenylphosphine-melatonin (TPP-MLT) was encapsulated in VCAM-1 antibodies-conjugated nanostructured lipid carriers (VCAM@TPP-MLT NLCs) for lung targeting delivery. VCAM@TPP-MLT NLCs could be preferentially internalized by inflammatory endothelial cells in lung tissues, and then the released TPP-MLT from NLCs effectively eliminated the excessive reactive oxide species (ROS) and ameliorated cell apoptosis. Overall, the results suggested that VCAM@TPP-MLT NLCs exhibited remarkable *in vitro* and *in vivo* therapeutic effect on ALI, and could be a promising and efficient strategy for the treatment of ALI.

## Introduction

Acute lung injury (ALI) is a common clinical syndrome characterized by intrapulmonary infiltration of a large amount of diffuse inflammatory cells, increased pulmonary microvascular permeability, severe edema in pulmonary alveoli and parenchyma as well as hyoxemia. Its deteriorated form is acute respiratory distress syndrome (ARDS), and may rapidly develop into multiple organ failure with poor prognosis and a fatality rate of 30%–50%. There is yet no safe and effective preliminary pharmacological intervention or strong treatment means for restraining the progress of ALI. Currently, the preliminarily applied therapeutic drugs for ALI include inhalation of vasodilators (nitric oxide, prostacyclin, etc.), glucocorticoid, non-steroidal anti-inflammatory drugs, and ketoconazole, but with no satisfactory therapeutic effect (Kaisers et al., 2004; Marik et al., 2011). Therefore, it has drawn more and more attention to develop a new approach for treating ALI based on its pathogenesis.

It has been found in recent studies that pulmonary vascular endothelial cells are the main target cells of ALI caused by invasive factors such as endotoxin and hypoxia (Barratt et al., 2014). ALI could lead to the change of activity and increase in permeability of pulmonary vascular endothelial cells, and also alter the serum level of specific proteins of vascular endothelial cells, such as angiotensin converting enzyme, tissue factor pathway inhibitor and von Willebrand factor (Ware et al., 2001; Oz and Lorke, 2021). The dysfunction and damage of endothelial cells can further aggravate leukocyte adhesion and transfer, imbalance of cause cytokines, and disorder of coagulation/anticoagulation regulation (Lou et al., 2019). The endothelial cells will be further damaged and conditions could be worsened if this process fails to be reversed. Therefore, it is of great research value and clinical significance to find an effective approach for ALI treatment starting from reversing the damage of pulmonary vascular endothelial cells.

Nanostructured lipid carriers (NLCs) are an effective strategy to improve drug biodistribution and reduce drug accumulation in non-target organs. NLCs is mainly made of safe lipid materials such as triglyceride, lecithin and wax. It has the advantages of good biocompatibility, easy mass production, high drug loading and low drug leakage (Zhai and Zhai, 2014). By modifying the surface of NLCs with ligands and antibodies, targeted delivery of drugs encapsulated in NLCs can be achieved. In addition, surface hydrophilicity modification with polyethylene glycol can reduce the clearance rate and protein binding rate, and prolong the half-life of drugs (Simone et al., 2009). As the main damaged organ in inflammatory and pathological state, the targeted therapy is of great significance. After intravenous administration, NLCs in the bloodstream can directly interfere with endothelium and contribute to rapid improvement of endothelial related diseases. It is interesting to note that the intravenously injected endothelium-targeted NLCs can competitively bind to the pulmonary endothelium. This advantage is mainly due to the following three reasons (Kaisers et al., 2004): Large endothelial surface area of pulmonary vessels (Marik et al., 2011); The “first pass effect” of venous blood in lung tissues (Barratt et al., 2014); The pulmonary artery is the first major capillary after intravenous administration, and its high volume and relatively slow blood perfusion rate promotes the integration of NLCs in blood flow with endothelial targets (Muzykantov, 2005).

VCAM-1 is a type of transmembrane glycoprotein mainly expressed on the surface of endothelial cell cavity. It is expressed in a low degree on resting endothelial cells but significantly higher when the endothelium is in the inflammatory pathological state (Papademetriou et al., 2014). Its expression was strongly correlated with inflammation (McClintock et al., 2008). Therefore, NLCs were modified by VCAM-1 monoclonal antibody, which is specifically associated with VCAM-1 receptor overexpressed in pulmonary endothelium under ALI pathological condition, to achieve lung targeting delivery of drugs and while reduce the potential systemic side effects.

**FIGURE 1:**
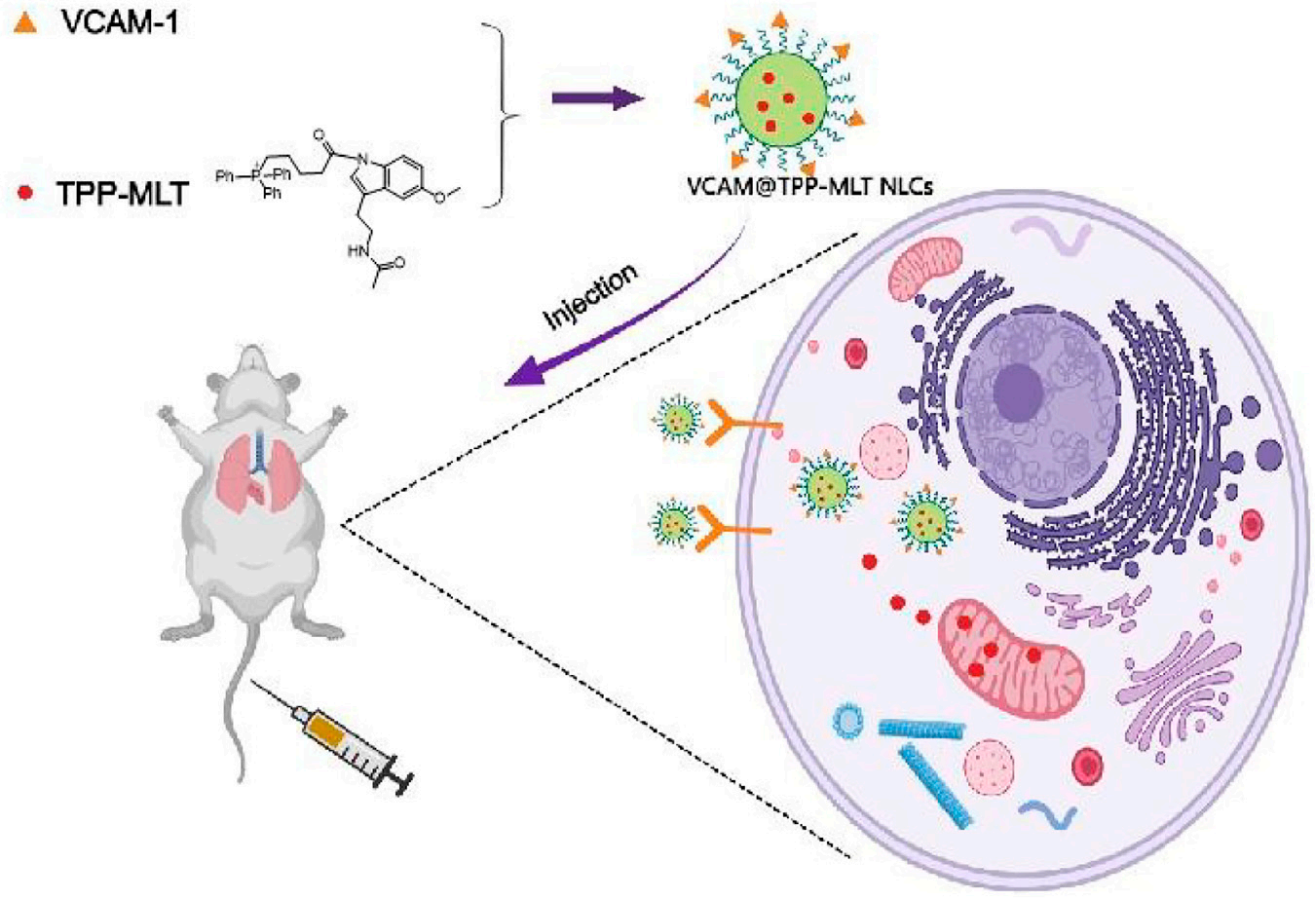
A schematic diagram of VCAM@TPP-MLT NLCs for the treatment of acute lung injury.

In this study, we developed a VCAM-1 antibodies-conjugated NLCs for active targeting delivery of drugs in inflammatory lung tissues of the ALI model. Melatonin (MLT) is extensively used for anti-inflammation, antioxidation and free radical scavenging. A mitochondrial targeting molecule triphenylphosphine (TPP) was conjugated to melatonin (TPP-MLT) to increase the distribution of MLT in intracellular mitochondria, which was detailed and characterized as our previous study (Hu et al., 2021). TPP-MLT was further encapsulated in VCAM-1 antibodies-conjugated NLCs (VCAM@TPP-MLT NLCs), and its physicochemical characteristics were evaluated. In addition, its distribution in inflammatory endothelial cells and lung tissues, and *in vitro* and *in vivo* therapeutic effects on ALI were assessed (Figure 1). The synthesis and characterization of TPP-MLT has been described in our previous study (Hu et al., 2021), and thus not included here.

## Results and discussion

### Preparation and characterization of VCAM@TPP-MLT NLCs

VCAM@TPP-MLT NLCs were prepared by the solvent diffusion method (Lu et al., 2017). A proportion of monostearic acid glyceride, polyethylene glycol_2000_-monostearate (PEG_2000_-SA), medium-chain triglycerides, NH_2_-PEG_2000_-SA and TPP-MLT were added to ethanol and heated to 60°C, followed by stirred under 400 rpm for 5 min. The obtained TPP-MLT NLCs were further mixed with N,N′-disuccinimidyl carbonate (DSC) to conjugated with VCAM-1 antibody. During the formation of NLCs, monostearic acid glyceride and medium-chain triglycerides in the organic phase rapidly solidified into hydrophobic cores of NLCs with the diffusion of organic solvents in the aqueous dispersion phase. The hydrophobic chain SA of PEG_2000_-SA was inserted into the core of NLCS, while the hydrophilic chain PEG_2000_ was distributed on the surface of NLCs to act as a hydrophilic modification, which helping to reduce the phagocytosis of NLCs by phagocytes and prolong the half-life of NLCs *in vivo*.

**FIGURE 2:**
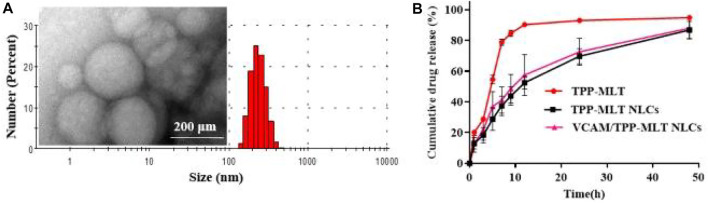
Characterization of VCAM@TPP-MLT NLCs. **(A)** Representative TEM image and DLS trace of VCAM@TPP-MLT NLCs. **(B)** Release behavior of VCAM@TPP-MLT NLCs in pH 7.4 PBS.

The particle size and particle size distribution of VCAM@TPP-MLT NLCs were presented in Figure 2A. VCAM@TPP-MLT NLCs showed homogeneously nano-structured particles in aqueous medium, and the hydrodynamic diameter and polydispersion index (PDI) of VCAM@TPP-MLT NLCs with different feeding ratio of TPP-MLT was presented in Table 1. The surface zeta potential of VCAM@TPP-MLT NLCs was negative, and the flocculation of the NLCs was promoted by adding acid to the dispersion solution to neutralize the negative charge on the surface of NLCs. TPP-MLT encapsulated in the NLCs was deposited with the NLCs by high-speed centrifugation, and then TPP-MLT dissolved in the aqueous dispersion phase was separated from VCAM@TPP-MLT NLCs. The entrapment efficiency (EE%) and drug loading (DL%) of NLCs were detected and calculated. With the increase of feeding amount, the DL% of TPP-MLT in VCAM@TPP-MLT NLCs almost did not increase, and therefore 10% feeding ratio of TPP-MLT was used in the following experiments. The corresponding EE% was 86.79 ± 2.81%, and DL% was 4.34 ± 0.14%.

**TABLE 1:** Characterization of VCAM@TPP-MLT NLCs with different feeding ratios of TPP-MLT.

Feeding ratio (%)	*d*_n_ (nm)	PDI	ζ (mV)	Drug loading (%)	Entrapment efficiency (%)
0	205.6 ± 20.9	0.158 ± 0.034	−23.1 ± 1.8		
5	221.4 ± 18.3	0.186 ± 0.049	−21.7 ± 2.1	4.34 ± 0.14	86.79 ± 2.81
10	248.1 ± 19.4	0.195 ± 0.058	−20.5 ± 2.2	10.95 ± 0.12	71.57 ± 0.77
15	253.2 ± 21.7	0.179 ± 0.063	−22.8 ± 1.6	11.73 ± 0.58	78.29 ± 3.86
20	255.8 ± 20.8	0.205 ± 0.048	−21.1 ± 1.9	11.93 ± 0.80	59.65 ± 4.00

The *d*_n_, PDI, and ζ values represent the hydrodynamic diameter, PDI, and zeta potential, respectively. The data represent the mean ± SD (n = 3).

The *in vitro* release behavior of VCAM@TPP-MLT NLCs was investigated using the dialysis method, free TPP-MLT and TPP-MLT NLCs as control (Wang et al., 2021). Figure 2B revealed that the free TPP-MLT released quickly, and almost all amount of TPP-MLT was released within approximately 12 h. In contrast, TPP-MLT loaded in the VCAM@TPP-MLT NLCs exhibited a sustained drug release behavior up to 48 h. The sustained drug release behavior of VCAM@TPP-MLT NLCs reduced drug leakage from NLCs in the bloodstream, and increased TPP-MLT accumulation in the lung tissues of ALI models. In addition, the release behaviors of VCAM@TPP-MLT NLCs and TPP-MLT NLCs were similar, suggesting that the conjugated VCAM-1 antibodies had little effect on the drug release.

### Safety and biocompatibility of VCAM@TPP-MLT NLCs

**FIGURE 3:**
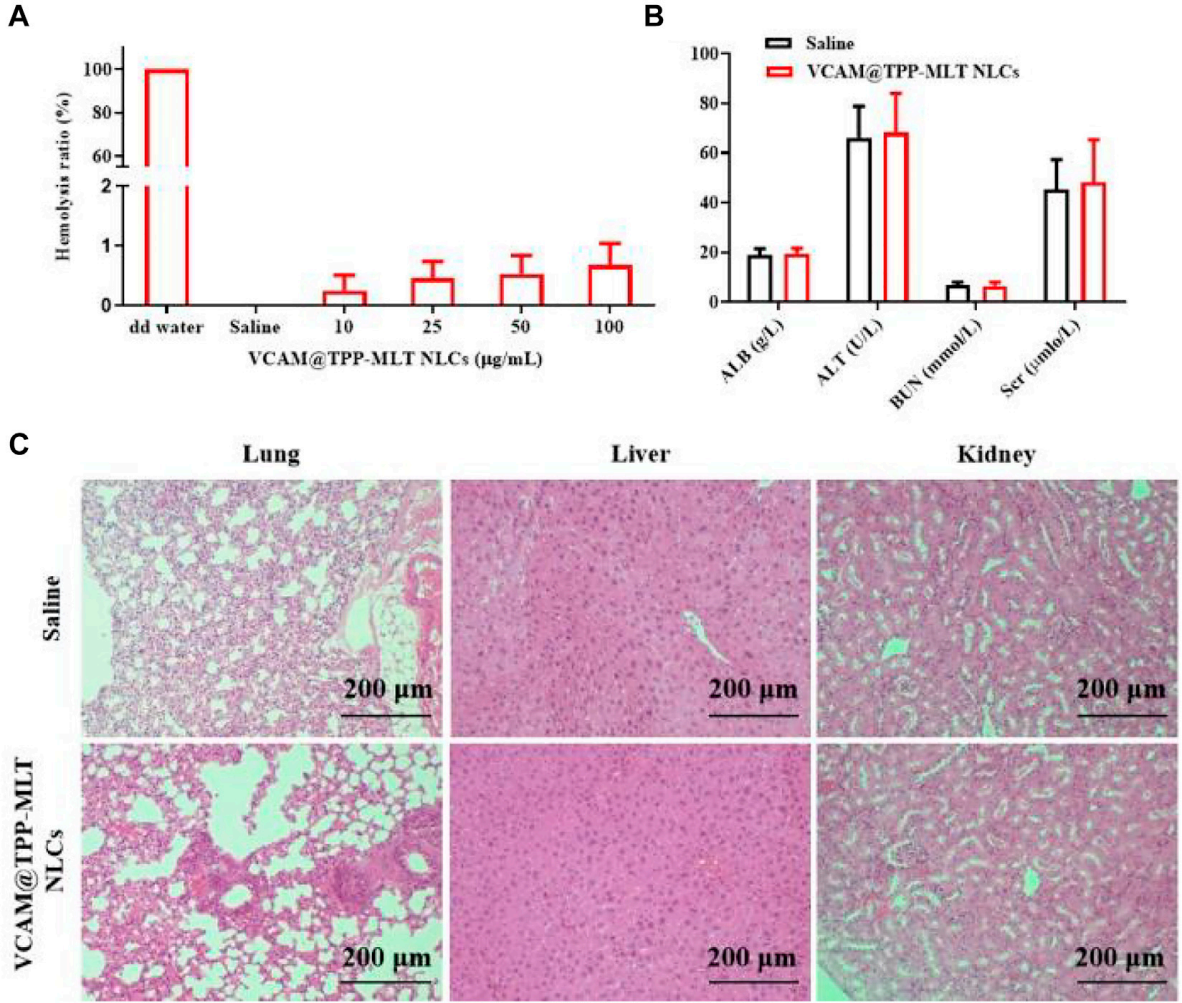
Safety and biocompatibility of VCAM@TPP-MLT NLCs. **(A)** Hemolysis ratios of VCAM@TPP-MLT NLCs. **(B)** The influences of VCAM@TPP-MLT NLCs on the liver and renal functions. The data represent the mean ± SD (*n* = 6). **(C)** Representative H&E staining images of liver, lung and kidney in mice treated with saline or VCAM@TPP-MLT NLCs.

VCAM@TPP-MLT NLCs were administrated via intravenous injection, and hemolysis test was performed to assess the safety of intravenous administration. As shown in Figure 3A, the hemolysis ratios of various concentration of VCAM@TPP-MLT NLCs were all lower than 1%, demonstrating the good hemocompatibility of VCAM@TPP-MLT NLCs. Healthy mice were given VCAM@TPP-MLT NLCs (TPP-MLT, 20 mg/kg) every 3 days for five consecutive times. After that, liver and renal functions were detected, and the results showed that VCAM@TPP-MLT NLCs had no effects on liver and renal functions (Figure 3B). The liver, lung and renal tissues were isolated, sectioned and stained by hematoxylin-eosin (H&E). As showed in Figure 3C, no obvious histopathological changes were observed in mice treated with VCAM@TPP-MLT NLCs.

### Internalization of VCAM-1 antibody-conjugated NLCs

**FIGURE 4:**
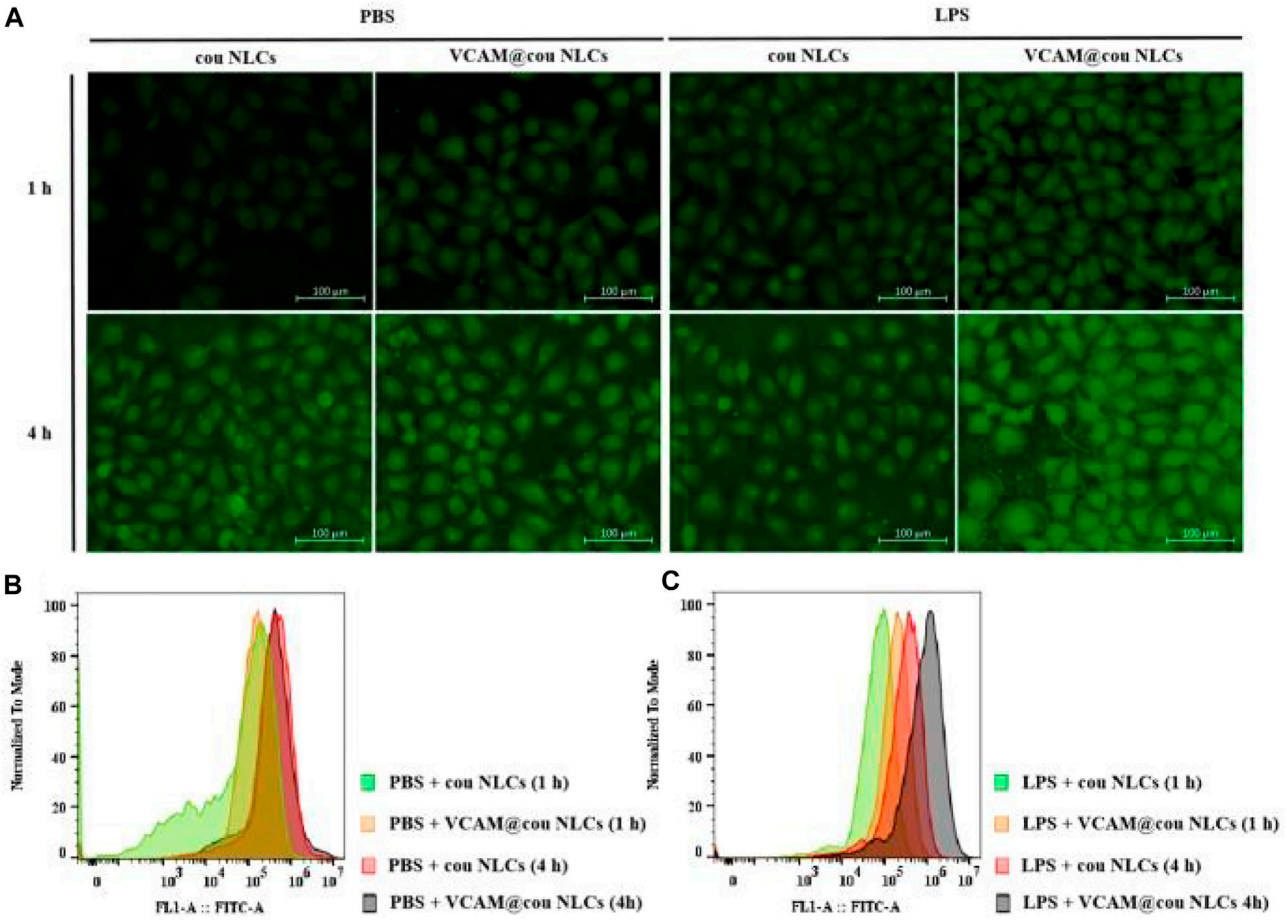
Targeted uptake of coumarin-6 (cou)-labelled VCAM-1 antibody-conjugated NLCs (VCAM@cou NLCs) by LPS-induced HUVECs. **(A)** Representative fluorescence images of VCAM@cou NLCs internalized by HUVECs after incubated for 1 and 4 h (Scale bar, 100 μm). **(B)** and **(C)** Flow cytometric analysis of VCAM@cou NLCs by HUVECs.

The increased expression of VCAM-1 receptors was observed in HUVECs during inflammation. Therefore, lipopolysaccharide (LPS) was firstly used to stimulate HUVECs, and then incubated with coumarin-6 (cou)-labeled VCAM-1 antibody-conjugated NLCs (VCAM@cou NLCs), cou NLCs as control. As shown in Figure 4A, the distribution of VCAM@cou NLCs in LPS-induced HUVECs was more than that in PBS-induced HUVECs after incubation of 1.0 and 4.0 h. In addition, the distribution of VCAM@cou NLCs was also more than that of cou NLCs in LPS-induced HUVECs, suggesting the increased distribution of VCAM@cou NLCs was associated with the conjugated VCAM-1 antibody. Meanwhile, HUVECs were collected for flow cytometric analysis. As show in Figure 4B, the fluorescence intensities of VCAM@cou NLCs and cou NLCs in HUVECs treated with PBS were similar at the same time. By contrast, the fluorescence intensities of VCAM@cou NLCs in HUVECs treated with LPS were higher than that of cou NLCs, both at 1 h and 4 h (Figure 4C).

**FIGURE 5:**
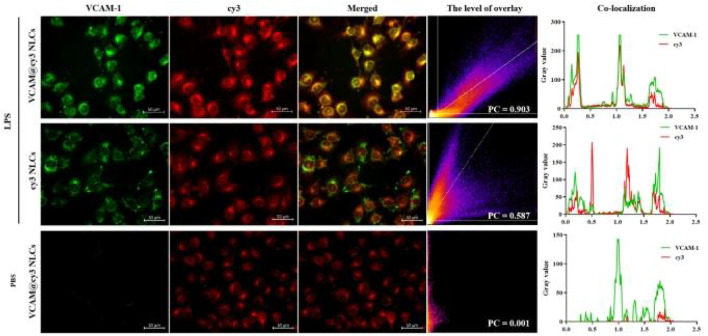
Colocalization analysis between cy3-labelled VCAM-1 antibody-conjugated NLCs (red) and VCAM-1 receptor (green), including level of overlay and Pearson’s correlation coefficient. (Scale bar, 50 μm).

Next, the internalization pathway of VCAM-1 antibody-conjugated NLCs was further firmed by fluorescence imaging technique, followed by a colocalization analysis using ImageJ software. As show in Figure 5, the green fluorescence signal represented the fluorescent secondary antibody-labeled VCAM-1 receptor, and red fluorescence signal represented the cy3-labeled NLCs with and without VCAM-1 antibody modification. It was observed that red fluorescence of VCAM-1@cy3 NLCs overlapped better with the green fluorescence of VCAM-1 receptor than that of cy3 NLCs in LPS-induced HUVECs (Pearson’s correlation coefficient, PC = 0.903 and 0.587 for VCAM-1@cy3 NLCs and cy3 NLCs, respectively). In addition, little expression of VCAM-1 receptor was observed in PBS-induced HUVECs (PC = 0.001). Pearson’s correlation coefficient is a common parameter in fluorescence colocalization analysis, and its value range is 1 to −1. 1 represents perfect positive correlation, and colocalization must occur at the same time. −1 represents that the two sides of negative correlation colocation must not appear at the same time. 0 represents random relationship, and both sides of the colocation appear randomly without correlation. The results demonstrated the binding behavior of VCAM-1 antibody-conjugated NLCs on the Megalin receptors, suggesting the transport of VCAM-1 antibody-conjugated NLCs in HUVECs is associated with the VCAM-1 receptors.

### Bio-distribution of VCAM-1 antibody-conjugated NLCs in ALI model

**FIGURE 6:**
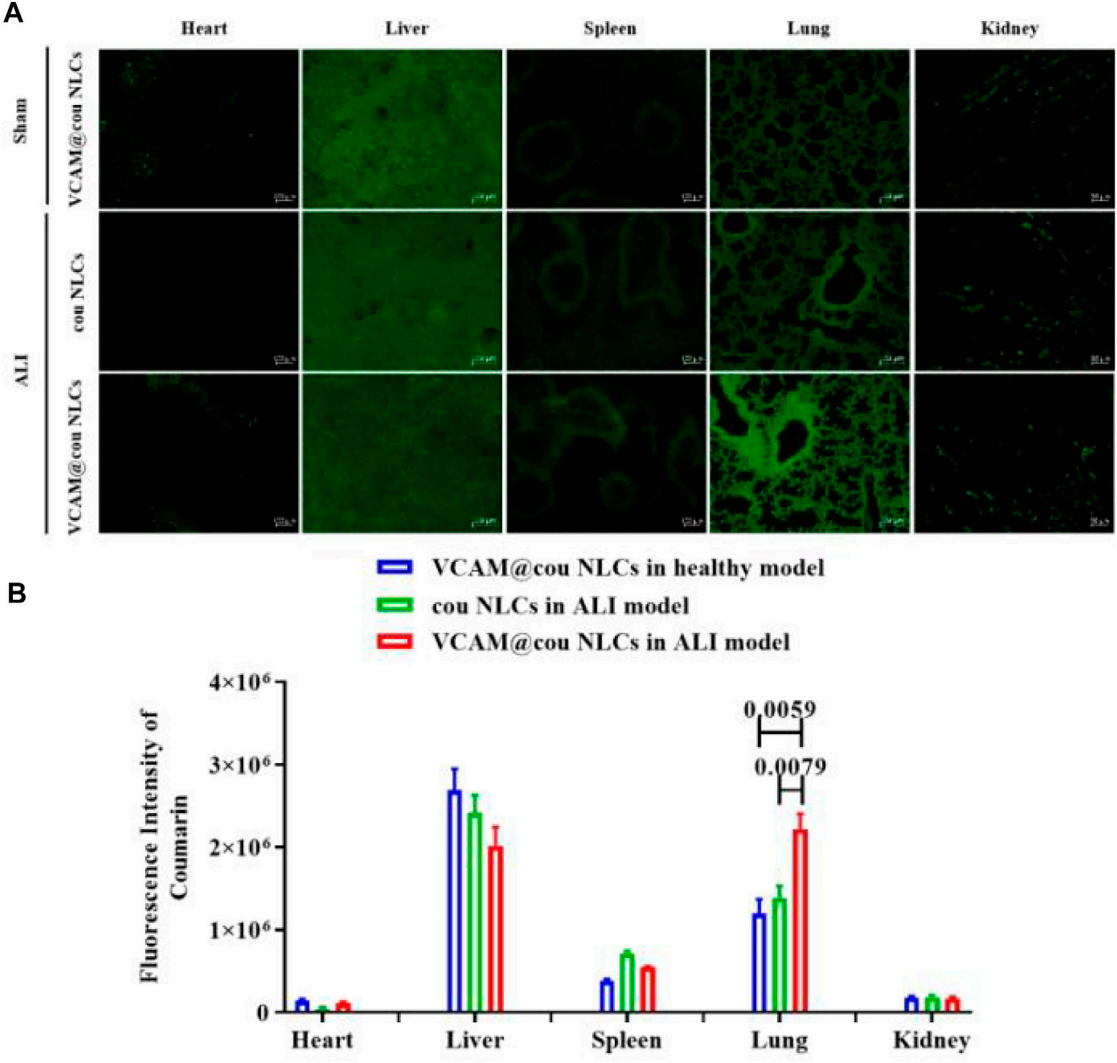
Bio-distribution of VCAM-1 antibody-conjugted NLCs. **(A)** Representative fluorescence images of VCAM@cou NLCs in each organ tissue (Scale bar, 100 μm). **(B)** Semiquantitative analysis of fluorescence intensity. (*n* = 3)

The bio-distribution of cou-labeled VCAM-1 antibody-conjugated NLCs (VCAM@cou NLCs) was evaluated in ALI mice. VCAM@cou NLCs was injected intravenously and the mice were executed at 6 h after administration. Tissue samples including heart, liver, spleen, lung, and kidney were isolated and fixed in 4.5% formalin for freeze sectioning, and the fluorescence signals of the drug in the tissues were observed under an inversed fluorescent microscope (Axio Observer 5, Zeiss). As shown in Figures 6A,B, the distribution of VCAM@ cou NLCs in the lung of ALI model is more than that of cou NLCs, and also more than that of VCAM@cou NLCs in lung of sham model, convincingly demonstrating the good lung distribution of VCAM-1 antibody-conjugated NLCs in ALI model.

### *In vitro* antioxidant activity of VCAM@TPP-MLT NLCs

**FIGURE 7:**
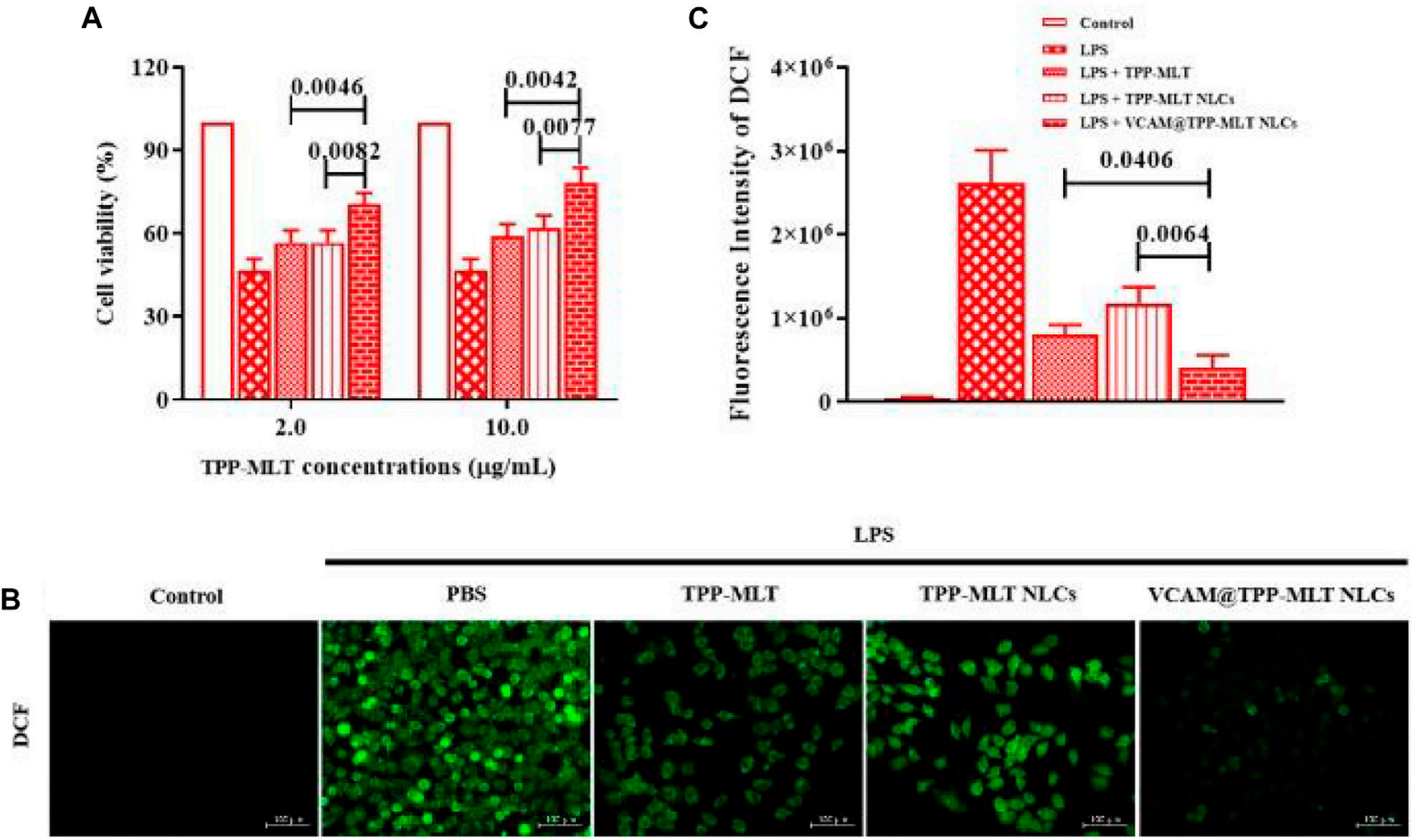
*In vitro* antioxidant activity of VCAM@TPP-MLT NLCs. **(A)** Cell viability of LPS-induced HUVECs after treated with VCAM@TPP-MLT NLCs, free TPP-MLT and TPP-MLT NLCs as control (*n* = 3). **(B)** Representative fluorescence images of intracellular ROS analysis with DCFH-DA in LPS-induced HUVECs (Scale bar, 100 μm). **(C)** Semiquantitative analysis of DCF fluorescence intensity in LPS-induced HUVECs.

The effects of VCAM@TPP-MLT NLCs on cell viability were investigated by MTT method (Mi et al., 2020). As shown in Supplementary Figure S1 and Figure 7A, the cell viability was approximately 40% after incubation with 15 μg/ml LPS for 24 h. Compared with MLT, TPP-MLT treatment significantly improved the cell viability of LPS-induced HUVECs at the concentration of 10 mM. After encapsulated in VCAM-1 antibody-conjugated NLCs, the distribution of TPP-MLT in the inflammation-damaged cells was increased. Consequently, the cell viabilities of LPS-induced HUVECs were significantly improved by VCAM@TPP-MLT NLCs in comparison to those by free TPP-MLT and TPP-MLT NLCs at two concentrations of TPP-MLT.

DCFH-DA probe has no fluorescence and can pass through the cell membrane freely. After entering the cell, DCFH-DA can be hydrolyzed to DCFH by the esterase in the cells, whereas DCFH does not penetrate the cell membrane, making the probe easy to load into the cells. Intracellular reactive oxygen species (ROS) can oxidize non-fluorescent DCFH to produce fluorescent DCF, which is positively related with intracellular ROS levels (Wang et al., 2016). Mitochondria are the main source of ROS and the main target of ROS. The damage of mitochondrial function produces amount of ROS, which leads to the mitochondrial membrane lipid peroxidation. MLT was covalently conjugated and then driven by TPP to enter the mitochondrial bilayer membrane, enabling mitochondrial-targeted antioxidant activity. Compared with MLT, TPP-MLT effectively reduced the ROS level in LPS-induced HUVECs (Supplementary Figure S2). After encapsulated in VCAM-1 antibody-conjugated NLCs, the distribution of TPP-MLT in the inflammation-damaged cells was increased. As shown in Figures 7B,C, ROS levels of LPS-induced HUVECs were reduced to varying degrees by free TPP-MLT, TPP-MLT NLCs and VCAM@TPP-MLT NLCs respectively, and VCAM@TPP-MLT NLCs was superior to TPP-MLT and TPP-MLT NLCs. VCAM@TPP-MLT NLCs could be preferentially internalized by the inflammation-damaged HUVECs, and then more MLT was distributed in mitochondria under the mediation of TPP to scavenge the overproduced ROS.

### *In vitro* anti-apoptosis activity of VCAM@TPP-MLT NLCs

**FIGURE 8:**
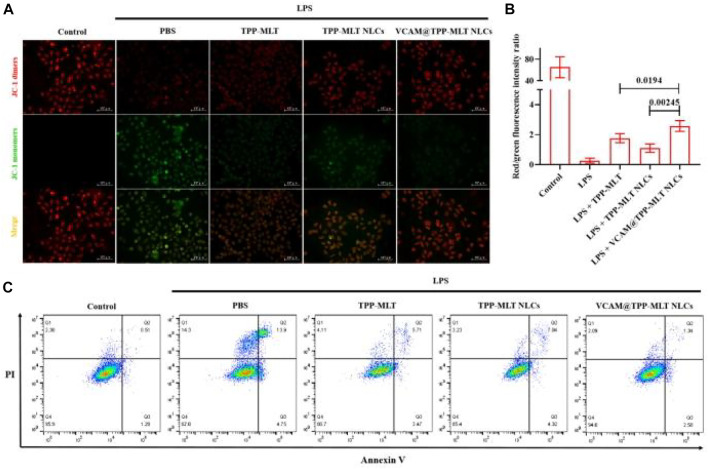
*In vitro* anti-apoptosis activity of VCAM@TPP-MLT NLCs. **(A)** Representative fluorescence images of JC-1 assay to measure mitochondrial membrane depolarization in LPS-induced HUVECs (Scale bar, 100 μm). **(B)** The ratio of fluorescence intensity of JC-1 aggregates and JC-1 monomers in **(A)**. **(C)** Flow-cytometry-based apoptosis assay by Annexin-FITC apoptosis kit.

High concentration of ROS can induce mitochondrial depolarization and decrease of mitochondrial membrane potential (MMP), which is one of the earliest events in the cascade reaction of apoptosis. The JC-1 is an indicator of MMP and commonly used to detect early apoptosis. In the healthy cells, JC-1 collects in the mitochondria due to the MMP, forming a polymer that emits red fluorescence. When cell apoptosis occurs, MMP is depolarized, JC-1 is released from mitochondria, red fluorescence is weakened and exists in cytoplasm as monomer, emitting green fluorescence. The change of MMP was indicated by the change of the ratio of green fluorescence to red fluorescence. When the ratio of green to red fluorescence increases, the MMP decreases. JC-1 assay was used to assess the effects of VCAM@TPP-MLT NLCs on the changes of MMP in LPS-induced HUVECs. Similar to the effect of TPP-MLT on the change of ROS level, it also obviously ameliorated the MMP of LPS-induced HUVECs in comparison to MLT (Supplementary Figure S3), which was closely associated with the increased distribution of MLT in mitochondria. After encapsulated in VCAM-1 antibody-conjugated NLCs, the effect of TPP-MLT was improved further. As shown in Figures 8A,B, the red/green fluorescence intensity ratio in LPS-induced HUVECs was enhanced to varying degrees after treatment. Compared with free TPP-MLT and TPP-MLT NLCs, the more significant change in the red/green fluorescence intensity ratio was observed in HUVECs treated with VCAM@TPP-MLT NLCs.

Annexin V-FITC/PI apoptosis detection kit was then used to assess the effects of VCAM@TPP-MLT NLCs on the apoptosis of LPS-induced HUVECs. As shown in Figure 8C, the apoptosis rate in normal cells was 0.51%. For the LPS-induced cells, the apoptosis rates for PBS group, TPP-MLT group, TPP-MLT NLCs group and VCAM@TPP-MLT NLCs group were 13.9%, 5.71%, 7.04% and 1.34%, respectively.

### *In vivo* pharmacodynamic evaluation on VCAM@TPP-MLT NLCs

**FIGURE 9:**
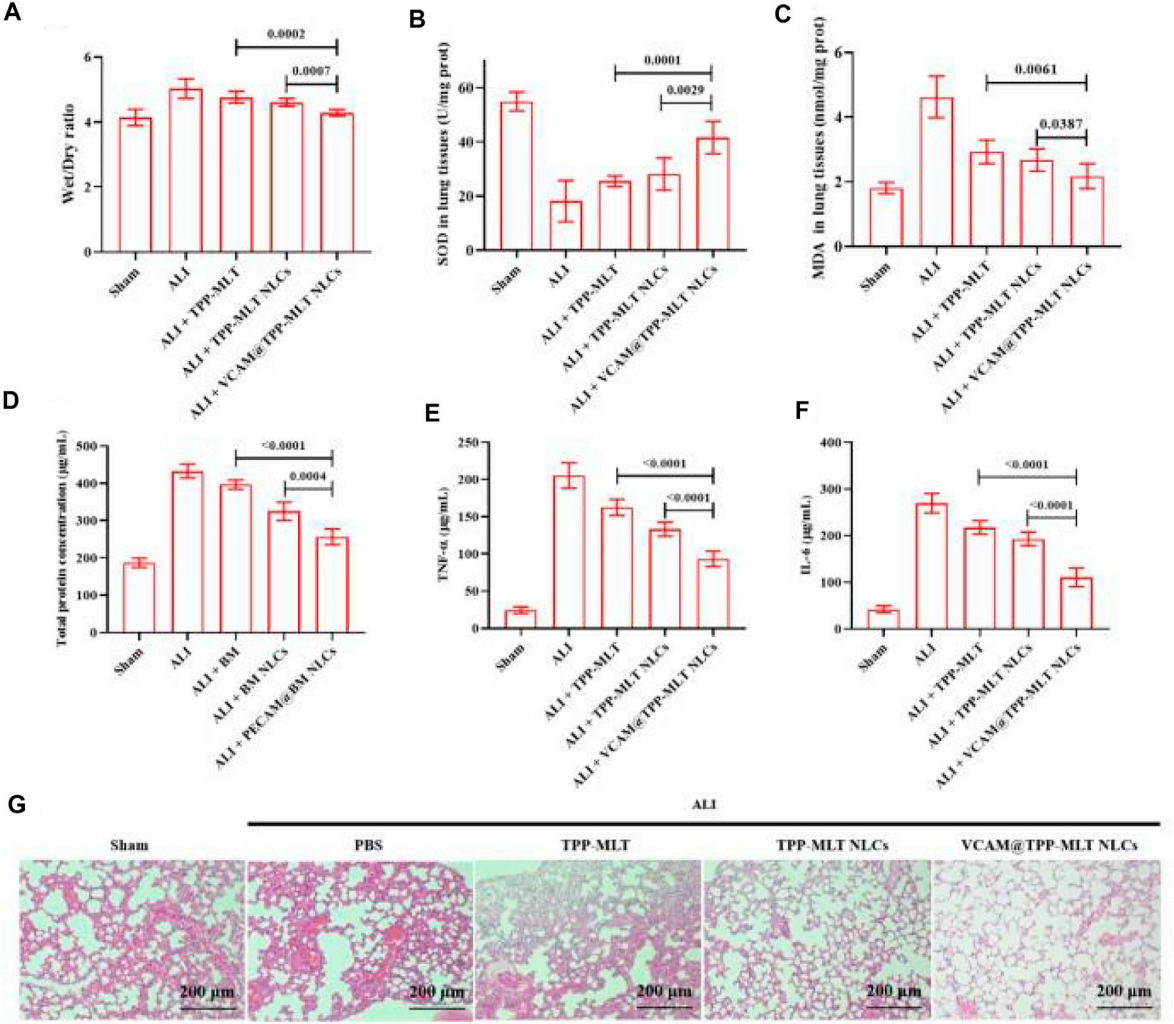
*In vivo* pharmacodynamic evaluation of VCAM@TPP-MLT NLCs. **(A)** Wet/dry ratio of lung tissues in ALI mice after treated with VCAM@TPP-MLT NLCs, TPP-MLT and TPP-MLT NLCs as control. **(B)** and **(C)** The effects of VCAM@TPP-MLT NLCs on the changes of SOD and MDA in the lung tissues of ALI mice. **(D)** The effects of VCAM@TPP-MLT NLCs on the changes of total protein concentration in BALF of ALI mice. **(E)** and **(F)** The effects of VCAM@TPP-MLT NLCs on the changes of proinflammatory cytokines (TNF-α and IL-6) in BALF of ALI mice. The data represent the mean ± SD (*n* = 6). **(G)** Representative H&E staining sections of lung tissues in ALI mice (Scale bar, 200 μm).

The therapeutic efficacy of VCAM@TPP-MLT NLCs was evaluated in ALI model. The ALI mice were intravenously injected with PBS, free TPP-MLT, TPP-MLT NLCs and VCAM@TPP-MLT NLCs (TPP-MLT, 5.0 mg/kg). After 24 h, the mice were sacrificed, and the lungs were collected for determination. Compared with the sham group, pulmonary edema was observed in the ALI group after LPS exposure, as reflected by wet/dry ratio of lung tissues. The pulmonary edema was significantly reduced in ALI mice treated with VCAM@TPP-MLT NLCs in comparison to those treated with free TPP-MLT and TPP-MLT NLCs (Figure 9A).

Oxidative stress response plays an important role in the development and progression of ALI. Among them, superoxide dismutase (SOD) and malondialdehyde (MDA) are the classic indicators of oxidative stress. In response to various inflammatory stimuli, lung endothelial cells can generate both nitric oxide and superoxide, which may react to form peroxynitrite that can nitrate and oxidize key amino acids in various lung proteins, and inhibit their functions. Enzymes such as SOD can provide cellular protection against damage from oxygen-derived free radicals. MDA is the breakdown product of polyunsaturated fatty acids by oxidation and reflects the damage caused by ROS. The markers of oxidative stress in lung tissues of ALI mice, including SOD and MDA, were detected to evaluate the antioxidant activity of VCAM@TPP-MLT NLCs. As shown in Figures 9B,C, the SOD in lung tissues of ALI mice was significantly depleted. Compared with free TPP-MLT and TPP-MLT NLCs, VCAM@TPP-MLT NLCs treatment significantly improved the levels of SOD in the lung tissues. In addition, VCAM@TPP-MLT NLCs also effectively reduced the abnormally increased MDA value in lung tissues of ALI mice.

Total protein concentration and proinflammatory cytokines (TNF-α and IL-6) in bronchoalveolar lavage fluid (BALF) were examined. As shown in Figure 9D, the increased total protein concentration was observed in ALI mice. Compared with TPP-MLT and TPP-MLT NLCs, VCAM@TPP-MLT NLCs treatment significantly reduced total protein concentration in BALF of ALI mice. The changes of TNF-α and IL-6 in BALF were examined via ELISA. As shown in Figure 9E, VCAM@TPP-MLT NLCs treatment significantly reduced TNF-α in BALF of ALI mice in comparison to those treated with TPP-MLT and TPP-MLT NLCs (both *p* < 0.0001). VCAM@TPP-MLT NLCs also effectively reduced the IL-6 level in BALF of ALI mice (Figure 9F).

The pulmonary histopathological changes of ALI mice were examined by hematoxylin and eosin (H&E) staining. The collected lung tissues were excised, fixed with 4% paraformaldehyde for 72 h, cut into 5 μm paraffin sections, stained with H&E and finally observed under light microscope (Primostar 3, Zeiss, Germany). As shown in Figure 9G, compared with the sham, the lung tissues of ALI mice showed the aggravated inflammatory cell infiltration, thickened alveolar walls, diffuse edema, reduced alveolar space and increased interstitial hyperemia. VCAM@TPP-MLT NLCs treatment significantly ameliorated the pulmonary histopathological changes in ALI mice, which of effect was better than those of free TPP-MLT and TPP-MLT NLCs.

## Discussion

ALI refers to acute diffuse lung injury caused by internal and external pulmonary pathogenic factors, and then develops into acute respiratory failure, featured by extensive pulmonary inflammation, endothelium injury, and osmotic pulmonary edema caused by increased pulmonary microvascular permeability. Therefore, effective improvement of pulmonary microvascular endothelium injury is the key to the effective treatment of ALI (Orfanos et al., 2004). ALI animal model was established by intratracheal injection of LPS in this study. The *in vivo* pharmacodynamic results demonstrated that VCAM@TPP-MLT NLCs effectively inhibited the pathological changes of ALI and alleviated pulmonary edema, as reflected by wet/dry ratio of lung tissues and histopathological changes.

It was reported that pulmonary vascular endothelial cells are not only the main target cells for the occurrence and development of ALI, but also the effector cells with the earliest pathological changes. Recent studies suggest that mitochondrial dysfunction plays an important role in the occurrence and development of ALI (Agrawal and Mabalirajan, 2016). Mitochondrial DNA is an important component that mediates the metabolic function of mitochondria and cells. Due to its naked appearance, lack of protective histones, imperfect damage repair mechanism, and close to the respiratory chain, it is extremely sensitive to ROS and is easy to be oxidized to cause mutations (Okamura and Pennathur, 2015). Damaged mtDNA can make nuclear transcription factors κB (NF-κB) activation, promote the production of inflammatory factors, and activate TLR9 receptors located in endosomes and plasma membranes in a variety of cells, especially vascular endothelial cells, leading to changes in its permeability and aggravating cell damage. Therefore, effective distribution of MLT in mitochondria of the damaged vascular endothelial cells is important for the treatment ALI. TPP is lipophilic cations, and the three benzene rings increase the surface area of the molecule and form a delocalized positive charge. The innermost part of the mitochondrial bilayer membrane is negatively charged, and the TPP can pass through the mitochondrial bilayer membrane. Because TPP covalently bind to MLT, MLT is driven by TPP and enters the mitochondrial bilayer membrane, enabling mitochondrial-targeted antioxidant activity. Compared with MLT, TPP-MLT effectively ameliorated cell viability, reduced intracellular ROS and improved MMP. On this basis, TPP-MLT was further encapsulated in VCAM-1 antibodies-conjugated NLCs, and VCAM@TPP-MLT NLCs showed the better *in vitro* and *in vivo* therapeutic effects on ALI benefiting from good distribution in lung tissues and endothelial cells.

It’s been reported that lung injury caused by LPS and other traumatic factors can lead to the release of oxygen free radicals, which can produce a large number of lipid peroxidation, further damage the structure and function of the cell membrane, leading to the increase of lung vascular permeability and exacerbation of pulmonary edema (Lu et al., 2021). In this study, VCAM@TPP-MLT NLCs effectively improved the levels of SOD in lung tissues, and reduced the level MDA, suggesting that VCAM@TPP-MLT NLCs can inhibit the oxidative stress response, improve the antioxidant capacity and alleviate ALI.

In summary, we successfully constructed VCAM@TPP-MLT NLCs by lipid material with good safety for the treatment of ALI. TPP-MLT encapsulated in NLCs were accurately delivered to inflammatory endothelial cells in lung tissues. The increased distribution of TPP-MLT in lung tissues effectively reduces the level of ROS in inflammatory cells, and ameliorates the progress of ALI, providing good potential for the treatment of ALI.

## Materials and methods

### Materials

4-Carboxybutyltriphenylphosphonium bromide (TPP) and melatonin were purchased from Aladdin Bio-chem Technology Co. Limited (Shanghai, China). Monostearin was purchased from Shanghai Chemical Reagent Co., Ltd. (Shanghai, China). Medium-chain triglycerides was gifted from Gattefosse (Saint-Priest, France). Anti-VCAM-1 antibody was purchased from Cell Signaling Technology (Boston, United States). Fetal bovine serum (FBS), DMEM media, PBS buffer, trypsin/EDTA, and penicillin-streptomycin were purchased from Hyclone Laboratories (Logan, United States). Lipopolysaccharide (LPS), NH_2_-PEG_2000_-SA and PEG_2000_-SA were purchased from Sigma-Aldrich (St Louis, MO, United States). SOD and MDA kits were purchased from Nanjing Jiancheng Bioengineering Institute (Nanjing, China). DCFH-DA was purchased from Beyotime Biotechnology (Beijing, China).

### Preparation and characterization of VCAM@TPP-MLT NLCs

VCAM@TPP-MLT NLCs were prepared by the solvent diffusion method. Firstly, 195 mg monostearic acid glyceride, 30 mg PEG_2000_-SA, 60 mg medium-chain triglycerides, 0.004 mg NH_2_-PEG_2000_-SA, TPP-MLT (various feeding ratio: 1, 5, 10, 15, and 20) were added to 1.5 ml ethanol and heated to 60°C, followed by stirred under 400 rpm for 5 min. After cooling to room temperature, the obtained TPP-MLT NLCs were mixed with 20 μL of 0.02 mg/mL N,N′-disuccinimidyl carbonate (DSC) solution (NH_2_-PEG_2000_-SA:DSC = 1:1, mol/mol) under 100 rpm for 4 h. Afterwards, 50 μg VCAM-1 antibody was added and sequentially stirred for another 4 h to obtain VCAM-1@TPP-MLT NLCs.

The particle size, particle size distribution and zeta potential of VCAM@TPP-MLT NLCs were detected by dynamic light scattering (DLS) using a Zetasizer (Anton Paar, Litesizer500). The morphologies of VCAM@TPP-MLT NLCs were examined by transmission electron microscopy (TEM) (Hitachi, H7650) with NLCs’ concentration of 0.1 mg/ml.

The TPP-MLT content was determined by UPLC-MS/MS as our previous report (Hu et al., 2021). The VCAM@TPP-MLT NLCs suspension was flocculated by adjusting pH value to 1.2 by hydrochloric acid. The VCAM-1@TPP-MLT NLCs was separated *via* centrifugal-ultrafiltration (MWCO 3000, Millipore Co.) for 15 min at 10,000 rpm. The TPP-MLT concentration in the supernatant was measured, and the EE% and DL% were calculated as following equations:

$$\text{EE\% }=\;\left({\text{M}}_{\text{a}}{-\text{M}}_{\text{s}}\right)/{\text{M}}_{\text{a}}\times 100\text{\%}$$ (1)

$$\text{DL\% }=\;\left({\text{M}}_{\text{a}}{-\text{M}}_{\text{s}}\right)/\left({\text{M}}_{\text{a}}{+\text{M}}_{\text{c}}{-\text{M}}_{\text{s}}\right)\;\times 100\text{\%}$$ (2)

here M_s_ is the mass of TPP-MLT in ultrafltrate. M_a_ is the mass of TPP-MLT added in the system. M_c_ is the mass of lipid materials added in system.

The *in vitro* drug release behavior from VCAM@TPP-MLT NLCs was evaluated by dialysis method using pH 7.4 PBS as dissolution medium. Briefly, VCAM@TPP-MLT NLCs (TPP-MLT, 1 mg) was put into dialysis bag (MW: 3.5 kDa) and dialyzed against PBS under sinking condition with horizontal shaking (60 rpm) at 37°C. At predetermined time points, the mediums were collected and replaced with fresh buffer solution. The TPP-MLT content in the release medium was detected by UPLC-MS/MS and computed.

### Cellular uptake

HUVECs were transferred to 24-well plate at a density of 5×10^4^ cells/well and cultured overnight. HUVECs were stimulated by LPS (15 μg/ml) for 24 h to establish the *in vitro* inflammatory cell model. Cells were incubated with VCAM@cou NLCs for 1.0 and 4.0 h, cou NLCs as control. At the end of the incubation, the supernatant was removed, and the cells were washed using PBS, followed by fixation with 4% paraformaldehyde (PFA) for 15 min at room temperature. Intracellular fluorescence was observed by an inversed fluorescent microscope (Axio Observer 5, Zeiss, Germany). After that, HUVECs were collected for flow cytometric analysis.

### Colocalization analysis on VCAM-1 receptors

HUVECs were transferred to a 12-well plate at a density of 5×10^4^ cells per well. After pretreatment with 15 μg/ml LPS for 24 h, VCAM@cy3 NLCs and cy3 NLCs were added for incubation of another 4 h. Then, HUVECs were fixed with methanol at −20°C, the VCAM-1 receptor was labeled by immunofluorescent staining, observed under an inversed fluorescent microscope and last conducted by ImageJ software for colocalization analysis.

### Cell viability

The cytotoxicity of VCAM@ NLCs was performed by MTT method on HUVECs. In brief, cells were seeded into 96-well plates with density of 1×10^4^ cells/well and allowed to adhere overnight, followed by incubation with 15 μg/ml LPS for 24 h. Then, TPP-MLT, TPP-MLT NLCs and VCAM@TPP-MLT NLCs at equal TPP-MLT concentration were added and incubated for another 24 h. After treatment, 20 μL 5 mg/ml MTT solution was added and incubated for another 4 h. And then, the medium was replaced with 100 μL DMSO and the absorbance at 570 nm of each well was recorded by a microplate reader (Thermo FC), followed by calculation for cell viability.

### DCFH-DA

The production of ROS was labeled using DCFH-DA fluorescent probes. HUVECs seeded in 24-well plate were allowed for adherence overnight, and then cells were treated with 15 μg/ml LPS for 24 h. Then, TPP-MLT, TPP-MLT NLCs and VCAM@TPP-MLT NLCs at equal TPP-MLT concentration were added and incubated for another 24 h. After treatment, DCFH-DA fluorescent probes were added and incubated for 20 min under 37°C. HUVECs were washed with cold PBS, fixed in 4.5% formalin and observed by an inversed fluorescent microscope.

### Mitochondrial membrane potential

JC-1 probe (E_x_ = 488 nm, E_m_ = 535 nm) was used to examine the changes of MMP in HUVECs. Cells were added into 24-well plate and cultured overnight. After incubated with 15 μg/ml LPS for 24 h, PBS, TPP-MLT, TPP-MLT NLCs, and VCAM@TPP-MLT NLCs at equal TPP-MLT concentration were added and incubated for another 24 h. After treatment, culture medium containing JC-1 probe (10 μg/ml) was added and incubated for 15 min. Then, cells were washed with serum-free cell culture medium and observed by an inversed fluorescent microscope.

### Establishment of ALI model

The Balb/c mice with 20–25 g body weight were supplied by Shanghai SLAC Laboratory Animal Co., Ltd. and have free access to enough food and water in a controlled environment. All surgical procedures and *in vivo* experiments were approved by Ningbo University Institutional Animal Care (NBU20220074) and Use Committee and were conducted in compliance with National Institutes of Health Guide for the care and use of laboratory animals. The mice were accustomed to the environment for at least 1 w before operation. ALI model was established by dripping LPS *via* exposure air tube: mice were anaesthetized 5% chloral hydrate (450 mg/kg) and fixed in a supine position. LPS (2 mg/kg) was intraperitoneally injected by microsyringe. The rapid erection of model mice after the completion of the infusion promoted the distribution of the infusion solution in the lung tissues.

### Bio-distribution of VCAM-1 antibody-conjugted NLCs

The bio-distribution of VCAM-1 antibody-conjugted NLCs was evaluated in ALI mice. VCAM@cou NLCs was injected intravenously and the mice were executed at 6 h after administration. Tissue samples including heart, liver, spleen, lung, and kidney were isolated, were fixed in 4.5% formalin for freeze sectioning, and the distribution fluorescence of the drug in the tissues was observed under an inversed fluorescent microscope.

### Wet/dry weight ratio of lung tissues

After treatment, the right lung was excised, drained and weighed to obtain wet weight, and then dried at 80°C for 48 h to obtain dry weight. The wet/dry ratio was calculated as an assessment of tissue edema.

### Histopathology examination

The left lower lung tissue was fixed with 4% paraformaldehyde for 72 h and cut into 5 μm paraffin sections, which were stained with H&E and observed under light microscope.

### Statistics

Data were processed using SPSS 14 statistical software. The measurement data were expressed as mean ± SD, and statistical differences were tested by one-way ANOVA followed by the analysis using *t*-test and post hoc Fisher’s test after the homogeneity test of variance. The tested differences were considered statistically significant if *p* < 0.05.

## Data Availability

The raw data supporting the conclusions of this article will be made available by the authors, without undue reservation.
