# Supplementary material for: Enhanced efficiency of melatonin by stepwise-targeting strategy for acute lung injury
Source: Front Bioeng Biotechnol. 2022 Sep 7;10:970743. doi: 10.3389/fbioe.2022.970743 (PMC9490046; doi:10.3389/fbioe.2022.970743)
Supplement: Supplementary file 1 [file DataSheet1.pdf]

Enhanced efficiency of melatonin by stepwise-targeting strategy for acute lung injury

Hongbo Wang[1], Jing Li[1], Jianbo Jin[1], Jingbo Hu[2,*], Chunlin Yang[1,*]

1. Department of pharmacy, Ningbo University affiliated Yangming Hospital, Yuyao 315400, China.

2. Faculty of Materials Science and Chemical Engineering, Ningbo University, Ningbo 315211, China.

* **Correspondence:** ycl0115@163.com (Chunlin Yang), hujingbo@nbu.edu.cn (Jingbo Hu)

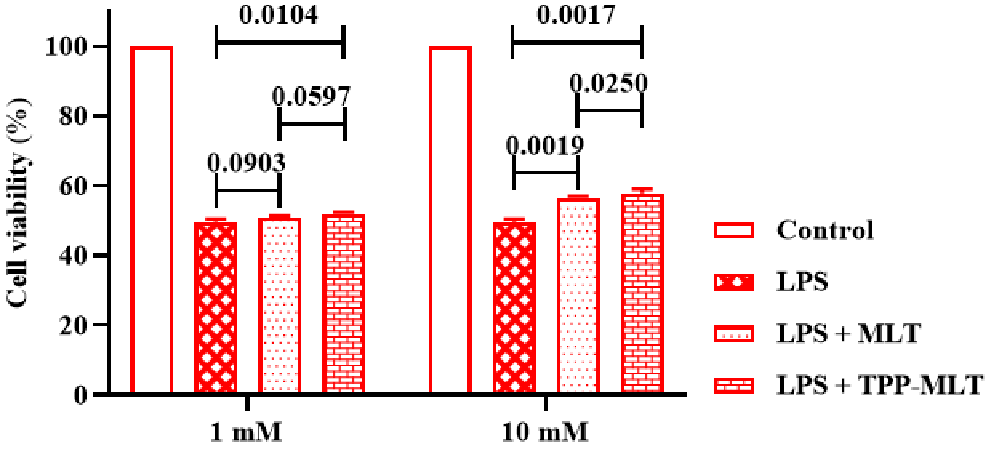

Figure S1. Cell viability of LPS-induced HUVECs after treated with TPP-MLT, MLT as control (n = 3).

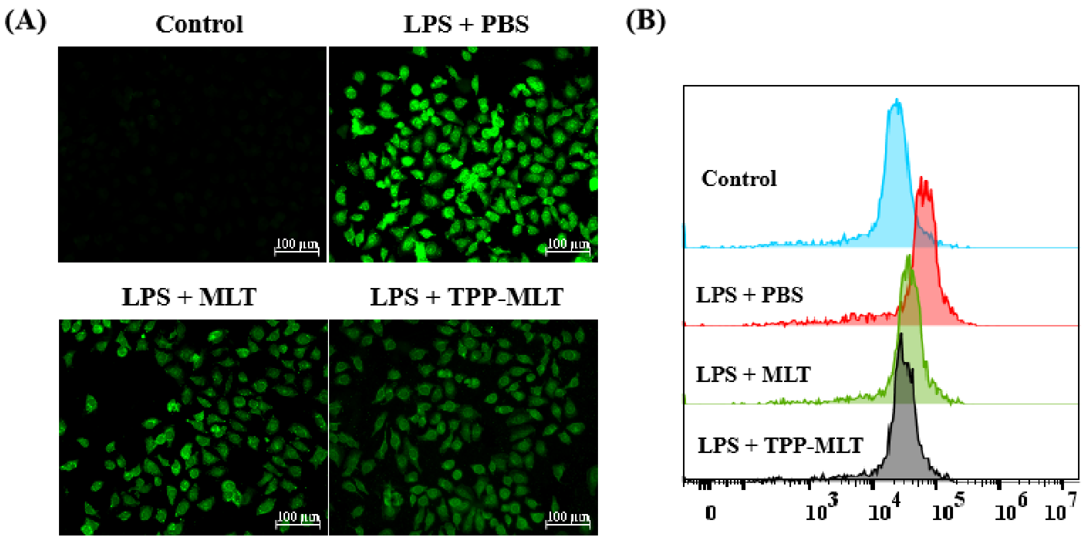

Figure S2. (A) Representative fluorescence images of intracellular ROS analysis in LPS-induced HUVECs after treated with MLT and TPP-MLT (Scale bar, 100 μm). (B) Flow cytometric analysis of intracellular ROS levels in LPS-induced HUVECs after treated with MLT and TPP-MLT. TPP-MLT (Scale bar, 100 μm).

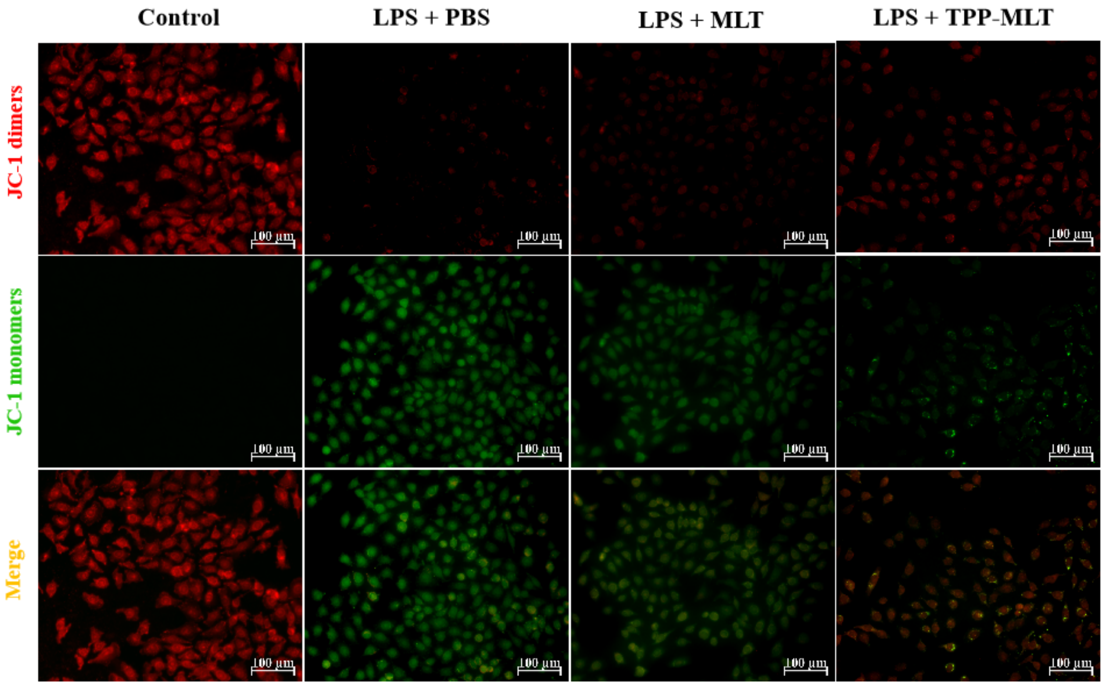

Figure S3. Representative fluorescence images of JC-1 assay to measure mitochondrial membrane depolarization in LPS-induced HUVECs after treated with MLT and TPP-MLT (Scale bar, 100 μm).
